# Explaining Chinese Reactions to COVID-19 During the Outbreak: A Systematic Illustration

**DOI:** 10.3389/fpubh.2021.727369

**Published:** 2021-12-08

**Authors:** Meng Yuan

**Affiliations:** School of Public Policy and Administration, Chongqing University, Chongqing, China

**Keywords:** COVID-19, China, trust, risk judgement, cognitive risk perception, affective risk perception, public attentions

## Abstract

**Objective:** This research attempts to explore systematically factors that influence public reactions during COVID-19 pandemic, including different measures of risk perceptions, public trust in different levels of governments, and attention to news.

**Methods:** This research uses a national stratified random sample of Chinese population and multiple linear regressions to explore the potential predictors of public reactions to coronavirus disease-2019 (COVID-19).

**Results:** This research found that the effects of attentions to news, provincial experience, trust in government, demographics, and political cultures on risk perceptions depend on measures of risk perceptions, risk judgments vs. cognitive vs. affective risk perceptions. Moreover, the effect of culture on trust in government is consistent across different levels of government, trust in local, provincial, and central governments; living in the epicenter of COVID-19 in China decreases trust in local/provincial government but not trust in central government; public attention to news can bring both positive (trust in government) and negative (negative affect) outcomes. Finally, it confirmed positive associations among risk perception, subjective knowledge, and attention to news.

**Conclusion:** The findings suggest challenges for risk communication.

## Introduction

### Background

Since COVID-19 first appeared in Wuhan in Hubei in China in January 2020, the virus has crossed borders to threaten the public health, economy, and regional stability in more than 200 countries and regions, made up of more than 124,535,520 of people infected and more than 2,738,876 people died as of 25 March 2021 (1). Existing research has suggested many reasons for the diversity of risk perception of COVID-19, such as informational sources (2), media content (3), COVID-19 experiences and psychological distance from crisis (4), knowledge and personal efficacy (5), trust in government and social media (6), time of imposing COVID-19 infection control policies (7), and demographics, such as age and gender (2–5, 7). Others focused on the consequences of risk perceptions, such as public concern of economic fallout after the COVID-19 outbreak (2), depression (5), public acceptance or adoption of measures against COVID-19, such as hand washing, mouth covering, and getting vaccinated (7, 8), public policy support, and infection rates (4, 6). A few of them has distinguished cognitive risk perception from affective risk perception and found that affective risk perception is positively correlated with while cognitive risk perception is negatively correlated with depression (4).

However, none of the research mentioned above has included the general measure of risk judgement, which is also a common and valid measurement of risk perception (9). More importantly, very few of them have provided a systematic picture of linkages among different kinds of public reactions to COVID-19, despite their value for informing future risk communication regarding novel infectious diseases. As an exception, Dryhurst et al. adopted a “holistic” approach to model the determinants of risk perception and found that personal experience, values, trust in government, science and medical professionals, knowledge, and personal and collective efficacy were all significant predictors of risk perceptions of COVID-19 (10). However, their research only focused on potential predictors of risk perception and ignored other kinds of public reactions to COVID-19.

To prevent overreliance on single-item measures of risk perception or selective measurement of either cognitive or affective dimensions of risk perception, this research taps into the cognitive and affective components of risk perception, risk judgment, and their correlations. More importantly, this research attempts to provide a systematic illustration of why people respond to a pandemic such as COVID-19 like they do. This research uses multiple linear regressions to explore potential predictors of different kinds of public reactions during the COVID-19 pandemic, including not only risk perceptions but also trust in government and attention to news.

### Theoretical Analysis

#### Potential Predictors of Risk Perceptions

Risk perception is a multidimensional conception. Most of the current research has measured the general level of perceived risk, labeled as risk judgment (9–11). Some research measured the emotional or affective risk perception that included concern, worry (12), negative affect (9, 13), and dread of risk (12, 14). Others focused on cognitive components of risk perception, such as probability or likelihood of realizing a risk (15), risk susceptibility (13), severity of consequences (16), and vulnerability (17).

Scholars have identified many factors influencing risk perception. Some of them suggested that the attributes of the threat itself can explain both affective risk perception and risk judgment (12, 15). Others combined the affective and cognitive components of risk to predict general risk judgment (16), but the causal relationship between cognitive and affective risk perceptions is still being debated [(9, 18) vs. (19, 20)]. This research will answer:

Research question 1a (RQ1a): How do affective and cognitive risk perceptions relate to risk judgment?

The characteristics of perceivers, such as knowledge, trust in risk managers (15, 21, 22), demographics (23), and proximity to risky areas (12, 13), can explain risk perception. However, the effect of trust in government on public fear or risk judgment varies by country [(10) vs. (24, 25)]. Moreover, risk perception research has found mixed results regarding the effect of knowledge on risk perception. For instance, positive effect on climate change (26) vs. negative effect on personal risk judgment in Ebola vs. positive effect on global risk in Ebola vs. no effect on concern about Ebola (27).

The social amplification of risk framework implies that concern about risk may be amplified when exposure to media coverage is increased (28, 29). Especially, previous research has found that the war metaphor was widely used by many social media networks, such as the People's Daily, to report on COVID-19, which may create a sense of urgency and increase psychological stress (30). However, the concern and perceived risk may be attenuated when people are exposed to information provided by their trusted risk managers (27).

Another group of scholars claimed that reactions to risk are not primarily due to properties of hazards themselves or individual variations in cognition but due to cultures. They used cultural theory (31, 32) to explain how culture influences risk perception in various kinds of hazards and risks, such as environmental risk (33, 34), vaccination (35), and Zika or Ebola epidemics (9, 15, 27, 36). Previous research has found that egalitarians perceived more risk of Ebola (15, 27, 36) than other cultures, while hierarchists are the opposite. Others provided evidence of the effect of culture (e.g., collectivistic vs. individualistic orientations) on public perceptions both within and between countries (37). Therefore, this research will answer:

RQ1b: How do COVID-19 provincial experience, trust in government, attention to news, subjective knowledge, cultural world views, and demographic factors relate to three types of risk perception (affective and cognitive risk perceptions, and risk judgment)?

#### Potential Predictors of Trust in Government

Some research challenged the causal model of trust and argued that high correlates between trust and risk perceptions do not necessarily mean that trust is the cause of risk perceptions. Eiser et al. proposed an associationist view of trust that, instead of the result, risk perceptions could be the determinant of trust (38). Others also found that affect heuristic, perceived risk, and acceptance of risk can, in turn, influence political trust (39).

Furthermore, the general public may react retrospectively. Research based on trust-as-evaluation tradition has provided evidence that the performance of government can influence political trust (40), especially the negative experience (41). When the COVID-19 outbreak started in Hubei province, the negative experience with COVID-19 may increase the distrust in government among residents of Hubei.

Research on political trust has suggested that modernization and political mobilization can influence political trust (42). Modernization can help people become “critical citizens” by increasing education and income (43). As a result, their political trust is reduced. Political mobilization refers to the exposure to government-controlled political propaganda and political education in China. Therefore, older generations in China and people who are more exposed to social medias often have a higher level of political trust (42). Research on public trust in government during COVID-19 found that older and healthy people trust more in their governments, whereas people who are better educated trust less, and these findings are supported across countries with different levels of economic condition and freedom (44). However, another research found that public trust in government is not influenced by demographic factors but by accessibility and trustworthiness of governmental communication (e.g., trustworthiness of information from mainstream media) (45).

Others used cultural theory to test the effect of culture on political trust (46). Individualists are more likely to distrust the government, viewing them as attempting to place limits on individual liberties. Individualists also have a lower degree of trust in the Centers for Disease Control (CDC) (15). On the contrary, hierarchists will trust the government, both because it represents authority, and because it has expertise. This research will answer:

RQ2: How do the three types of risk perception, COVID-19 provincial experience, attention to news, cultural world views, and demographic factors relate to trust in government?

#### Potential Predictors of Attention to News

Attention to news can be, in turn, influenced by risk perception and perceived knowledge level. The risk information seeking and processing (RISP) model (47) implies that information insufficiency and risk perceptions are determinants of information seeking. Specifically, affective response is seen to be instantaneous and guide information processing: in an uncertain situation, a higher level of health risk perception (48, 49) and knowledge (50, 51) motivated people to seek more information about the source of risk. However, it is also possible that individuals who are more knowledgeable are more likely to engage with information about the source of risk (27). In addition, people living in risky areas may pay more attention to the information about risk (13). Therefore, this research will answer:

RQ3a: How do the three types of risk perception, COVID-19 provincial experience, and subjective knowledge relate to attention to news?

## Methods

### Survey Participants

The survey was conducted from February 17 to March 1, 2020 by Zhongnan University of Financial and Economics in China, 3 weeks after Wuhan announced the lockdown. Data included 2,863 valid responses from 32 provinces in China (including five autonomous regions and four municipalities). Respondents came from the Tencent Questionnaire platform, a stratified random sample (gender, age, education) of Chinese adults. The respondents are 61% female; about 55% of them are younger than 30, and about 10% of them are older than 50; about 9% of the respondents have a high school degree or less education. Survey instruments are listed in Appendix A.

### Variables

The measurements of all variables are summarized in Table 1. The measurements are mainly adapted from existing research on public health crisis (4, 5, 12, 13, 15, 24, 52).

**Table 1:** Measurements of dependent variables and survey of public reactions to coronavirus disease-2019 (COVID-19), China, 2020.

**Variables**	**Measurements**
Risk judgement [Cronbach's alpha (α) = 0.62]	*Risk to self*: How much risk does the coronavirus pose to you or your family? *Risk to China*: How much risk does the coronavirus pose to China? *Risk to global*: How much risk does the coronavirus pose to the world? (1 = No risk, 2 = Little risk, 3 = Slight risk, 4 = Moderate risk, 5 = High risk, 6 = Very high risk)
Affective Risk perception^a^ (α = 0.47)	*Dread of risk:* How much you feel dread of coronavirus? (1 = No dread, 2 = Slight dread, 3 = Some dread, 4 = Moderate dread, 5 = High dread, 6 = Very high dread) *Positive affect: H*ow does considering the coronavirus make you feel, from very bad to very good? (1 = Very bad, 2 = Somewhat bad, 3 = Slightly bad, 4 = Neither good nor bad, 5 = Slightly good, 6 = Somewhat good, 7 = Very good)
Cognitive Risk perception: Risk susceptibility^b^ (α = 0.47)	Please answer whether you agree or disagree with the statements? *Risk susceptibility -my area:* My area is one of the places that is very likely to be affected by the coronavirus. *Risk susceptibility -other area:* The coronavirus mainly affects areas far from where I live in. *Personal risk susceptibility:* The coronavirus is most likely to have a big impact on people like me. (1 = Strongly disagree, 2 = Disagree, 3 = Slight disagree, 4 = Slight agree, 5 = Agree, 6 = Strongly agree)
Cognitive Risk perception: Likelihood of a future outbreak	How likely you think there will be another large outbreak of coronavirus in China in the next 5 years? (1 = Not at all likely, 2 = Somewhat unlikely, 3 = Somewhat likely, 4 = Very likely)
Trust in government (α = 0.81)	*Trust in local government:* Please rate how much you trust your local government (municipal or lower level government) to protect people from the coronavirus? *Trust in provincial government:* Please rate how much you trust your provincial government to protect people from the coronavirus? *Trust in central government:* Please rate how much you trust your central government to protect people from the coronavirus? (1 = No trust at all, 2-Slight trust, 3 = Moderate trust, 4 = High trust).
Attentions to news	How closely are you following news about the coronavirus infections in China? (1 = Not at all, 2 = Once a month, 3 = Once a week, 4 = Many times a week, 6 = Many times a day).
Subjective knowledge	How much do you know about the coronavirus? (1 = Never heard of it, 2 = Heard of it but don't know any more, 3 = Know about it in general but not details, 4 = Know some details about it, 5 = now a lot of details about it, 6 = I am an expert on COVID-19).
Demographic variables	*Age:* 1 = under 20, 2 = 20–9 years old, 3 = 30–39 years old, 4 = 40–49 years old, 5 = 50–59 years, 6 = over 60 years old) *Gender:* 1 = Male, 0 = Female *Education:* 1 = junior high school and below, 2 = high school, 3 = 3 years college, 4 = undergraduate, 5 = master's degree, 6 = PhD *Household income:* 1 = below 50,000, 2 = 50,000–100,000, 3 = 100,000–200,000, 4 = 200,000–500,000, 5 = 500,000–1,000,000, 6 = more than 1,000,000
COVID-19 provincial experience	A dummy variable of (1 = Yes, 0= No) is constructed from whether the respondent is a residence of Hubei province where the epicenter of COVID-19 outbreak in China.
Cultures	Each cultural index of four types of culture (*Individualism, Hierarchy, Fatalism*, and *Egalitarianism)* is measured by the sum of scores of three survey statement for each of four types of culture.

a *Cronbach's alpha of two variables of affective risk perception is lower than 0.5. Therefore, they are used separately in statistical models*.

b *The value of Cronbach's alpha is lower than the unacceptable threshold of 0.5. Therefore, these three variables are tested separately*.

### Variables

#### Dependent Variables

First, this research includes three sets of risk perception variables: general *risk judgment*, affective components of risk, namely, *dread of risk* and *positive affect*, and cognitive components of risk, namely, *risk susceptibility* and *likelihood of a future outbreak*. Second, the survey also asked about two other topics of interest, namely, *trust in government* and *attention to news*. These variables are used both as dependent and independent variables. For variables measured by multiple survey items, these survey items are tested both separately and combined as indices if Cronbach's alpha reached the.6 minimum (53). Principal component analysis was performed to create the indices.

#### Independent Variables

Many independent and control variables are included in all models. They are *subjective knowledge*; demographic variables namely *age, gender, education*, and *household income*; *COVID-19 provincial experience*; and four cultural indices namely, *individualism, hierarchy, fatalism*, and *egalitarianism*.

### Data Analysis Procedures

Aside from reporting frequencies, this research conducts multiple linear regression analyses for each of dependent variables. The rationality of linear regression is based on a series of assumptions. Normal P-P plots and scatterplots of the residuals indicated no violation of assumptions of normality and homoscedasticity. The correlation matrix (Appendix B) shows a medium level of correlation (ρ = −0.47) between *trust in provincial government* and *COVID-19 provincial experience*, and a medium to high level of correlations among the three trust variables, which indicates a multicollinearity problem. Therefore, the three trust variables were combined as t*rust in government* index when they were included as independent variables. Moreover, *risk to China* has a high correlation (ρ = 0.6) with *risk to global*. Therefore, *risk judgment* index was used when risk judgement variables were included as independent variables.

## Results

### Descriptive Statistics

Descriptive statistics of continuous variables is displayed in Appendix C.

On average, we see a relatively high level of risk judgment and negative affect (e.g., mean of affective feeling of COVID = 1.59, between “very bad” and “somewhat bad”). More than half (about 58%) agree or strongly agree that their area is one of the places that are very likely to be affected, and about 57% believe that there is somewhat or very likely there will be another large outbreak of COVID-19 in China within the next 5 years. However, we do not see a national panic, because the dread of COVID-19 is not that high (the mean of dread of risk = 3.42, between “some dread” and “moderate dread”), and <24% of them feel dread or very dread of COVID-19.

Moreover, the data show that perceived risk to China is higher than perceived risk to themselves and the world (mean value of risk to self = 3.65, risk to China = 4.92; risk to global = 4.65). About 74 and 59%, separately, rated risk to China and global as high or very high, but only 27% rated risk to self as high or very high.

By *t*-tests for independent samples, respondents who live in the epicenter of COVID-19 scored significantly higher on risk to self (residents of Hubei: M = 4.18, SD = 0.04; non-residents of Hubei: M = 3.43, SD = 0.02; *p* < 0.001) but significantly higher on risk to global (residents of Hubei: M = 4.49, SD = 0.02; non-residents of Hubei: M = 4.9, SD = 0.04; *p* < 0.001) and have no difference on risk to China. The residents of Hubei also scored significantly higher on dread of risk (M = 3.53, SD = 0.04; non-residents of Hubei: M = 3.37, SD = 0.03; *p* < 0.001) and risk susceptibility to their area (M = 5.17, SD = 0.04; non-residents of Hubei: M = 4.12, SD = 0.03; *p* < 0.001) and self (M = 4.03, SD = 0.04; non-residents of Hubei: M = 3.77, SD = 0.03; *p* < 0.001), but did not differ from the non-residents of Hubei on the likelihood of a future outbreak.

Compared to trust in central government (Mean = 3.47, between “moderate trust” and “high trust”), trust in local government (Mean = 2.99, between “slight trust” and “moderate trust”) and provincial government (Mean = 2.3) are lower. About 6% reported to not trust local or provincial government at all, but only about 1% of them reported to not trust central government. This is consistent with previous evidence of the phenomenon of hierarchical trust that people in China are more trusting of the central government than they are of the local government (54, 55). Another possibility of this trust differential in China is the respondents' self-censorship with regard to distrust for central leaders and incentives to hide their true political preferences in authoritarian regimes (56).

On average, the public paid high level of attention to COVID-19 (Mean = 5.42, between “many times a week” and “many times a day”), with more than 50% of them following news about COVID-19 many times a day.

### Multivariate Analysis

As Table 2 shows, the three measures of risk judgment are associated with dread of risk, risk susceptibility-my area, and attention to news. However, the other independent variables are associated with the three measures of risk judgment in different ways. Specifically, risk to self is associated with personal risk susceptibility, age, COVID-19 provincial experience, low risk susceptibility of other area, and low fatalism. Risk to China is associated with risk susceptibility of other area, likelihood of a future outbreak, trust in government, egalitarianism, hierarchy, negative affect, younger and less educated individuals, and no COVID-19 provincial experience. Risk to global has a similar pattern of correlation, except that it is associated with subjective knowledge but not associated with age.

**Table 2:** Linear regressions of risk judgement and survey of public reactions to COVID-19, China, 2020.

	**Risk to self**	**Risk to China**	**Risk to global**	**Risk judgement index**
**Affective risk perception**				
Dread of risk	0.28***	0.12***	0.11***	0.26***
Positive affect	−0.01	−0.16***	−0.14***	−0.22***
**Cognitive risk perception**				
Risk susceptibility-my area	0.27***	0.11***	0.04**	0.21***
Risk susceptibility-other area	−0.04*	0.05***	0.05***	0.06**
Personal risk susceptibility	0.06**	0.01	0.02	0.04*
Likelihood of a future outbreak	0.05	0.05*	0.12***	0.13***
Trust in government	−0.01	0.02*	0.08***	0.07***
Attentions to news	0.05*	0.08***	0.06**	0.12***
Subjective knowledge	0.05	0.01	0.05*	0.05
**Demographics**				
Gender	0.06	0.02	0.05	0.07
Age	0.11***	−0.06***	−0.01	−0.01
Education	−0.06*	−0.04*	−0.10***	−0.11***
Household income	0.01	−0.03	−0.03	−0.03
**Cultures**				
Individualism	−0.00	−0.00	−0.01	−0.01
Egalitarianism	−0.02	0.01*	0.01*	0.01
Fatalism	−0.02*	−0.01	0.00	−0.01
Hierarchy	0.01	0.02**	0.02**	0.03***
COVID-19 provincial experience	0.38***	−0.09*	−0.12*	0.00
Constant	0.92***	3.46***	3.26***	−3.34***
F significance	63.81***	34.60***	27.26***	46.73***
R^2^	0.29	0.18	0.15	0.25
R^2^ Adjusted	0.28	0.17	0.14	0.24

* *p < 0.05*;

** *p < 0.01*;

*** *p < 0.001*.

As Table 3 shows, dread of risk and positive affect are correlated negatively. Both dread of risk and negative affect are associated with risk judgment index and risk susceptibility of the respondents' area. Moreover, dread of risk is also associated with risk susceptibility of other area, likelihood of a future outbreak, younger individuals, and females (vs. males), while positive affect is also associated with less attention to news and low egalitarianism.

**Table 3:** Linear regressions of affective risk perception and survey of public reactions to COVID-19, China, 2020.

	**Dread of risk**	**Positive affect**
Risk judgement index	0.23***	−0.07***
**Affective risk perception**		
Dread of risk	—	−0.16***
Positive affect	−0.44***	—
**Cognitive risk perception**		
Risk susceptibility-my area	0.06**	−0.03**
Risk susceptibility-other area	−0.01	0.00
Personal risk susceptibility	0.12***	−0.00
Likelihood of a future outbreak	0.16***	0.01
Trust in government	−0.04	−0.01
Attentions to news	−0.01	−0.10***
Subjective knowledge	0.01	0.04**
**Demographics**		
Gender	−0.26***	0.02
Age	0.05*	−0.02
Education	−0.01	−0.02
Household income	0.00	0.02
**Cultures**		
Individualism	−0.00	0.01
Egalitarianism	−0.00	−0.01**
Fatalism	0.00	0.00
Hierarchy	0.02	0.00
COVID-19 provincial experience	−0.03	0.02
Constant	3.12***	2.75***
F significance	48.55***	32.34***
R^2^	0.25	0.17
R^2^ Adjusted	0.25	0.16

* *p < 0.05*;

** *p < 0.01*;

*** *p < 0.001*.

As Table 4 shows, all cognitive risk perception variables are positively associated with each other. Risk susceptibility-my area and personal risk susceptibility are associated with risk judgment index, dread of risk, attention to news, and low trust in government. Moreover, perceived risk susceptibility of area where the respondents live in is also associated with education, household income, egalitarianism, COVID-19 provincial experience, negative affect, and low fatalism, while personal risk susceptibility is also associated with males (vs. females), fatalism, and less educated. Perceived risk susceptibility of other area is associated with risk judgment index, females, and non-residence of Hubei province (vs. residence). Likelihood of a future outbreak is associated with risk judgement index, dread of risk, age, fatalism, low trust in government, low subjective knowledge, low hierarchy, and non-residents of Hubei province.

**Table 4:** Linear regressions of cognitive risk perception and survey of public reactions to COVID-19, China, 2020.

	**Risk susceptibility-my area**	**Risk susceptibility-other area**	**Personal risk susceptibility**	**Likelihood of a future outbreak**
Risk judgement index	0.18***	0.06***	0.04*	0.05***
**Affective risk perception**
Dread of risk	0.06**	−0.01	0.13***	0.06***
Positive affect	−0.09**	0.01	−0.00	0.01
**Cognitive risk perception**
Risk susceptibility-my area	—	0.10***	0.26***	0.05***
Risk susceptibility-other area	0.09***	—	0.17***	0.05***
Personal risk susceptibility	0.23***	0.18***	—	0.06***
Likelihood of a future outbreak	0.13***	0.15***	0.16***	—
Trust in government	−0.05***	0.03	−00.05**	−0.04***
Attentions to news	0.06*	−0.03	0.11***	−0.01
Subjective knowledge	0.01	0.02	−00.03	−0.03*
**Demographics**
Gender	−0.06	−0.10*	0.21***	0.05
Age	−0.02	0.02	0.03	0.06***
Education	0.12***	0.00	−0.10***	0.02
Household income	0.07***	0.04	0.02	0.01
**Cultures**
Individualism	0.01	−0.01	−0.00	0.00
Egalitarianism	0.02*	−0.01	0.00	0.00
Fatalism	−0.02*	0.02	0.06***	0.02***
Hierarchy	−0.00	0.01	−0.00	−0.01*
COVID-19 provincial experience	0.89***	−0.68***	0.02	−0.08*
Constant	1.53***	2.25***	0.43***	1.03**
F significance	77.51***	24.01***	49.93***	23.82***
R^2^	0.33	0.13	0.24	0.13
R^2^ Adjusted	0.32	0.12	0.24	0.12

* *p < 0.05*;

** *p < 0.01*;

*** *p < 0.001*.

Four measures of trust in government are all associated with risk judgment index, attention to news, hierarchy, low perceived risk susceptibility to self, low likelihood of a future outbreak, less educated, low individualism, and low fatalism (see Table 5). In addition, trust in local and provincial governments are also associated with low dread of risk, low perceived risk susceptibility of the area where respondents live in, and non-residents of Hubei province; trust in provincial government is also associated with perceived risk susceptibility of other area; trust in central government is also associated with low household income.

**Table 5:** Linear regressions of trust in government and survey of public reactions to COVID-19, China, 2020.

	**Trust in local government**	**Trust in provincial government**	**Trust in central government**	**Trust in government index**
Risk judgement index	0.04***	0.05***	0.02*	0.08***
**Affective risk perception**
Dread of risk	−0.03*	−0.03*	−0.00	−0.04
Positive affect	−0.03	−0.01	0.02	−0.02
**Cognitive risk perception**
Risk susceptibility-my area	−0.04***	−0.04***	0.00	−0.06**
Risk susceptibility-other area	0.02	0.03*	0.01	0.04
Personal risk susceptibility	−0.03**	−0.03*	−0.02*	−0.06**
Likelihood of a future outbreak	−0.04*	−0.08***	−0.08***	−0.14****
Attentions to news	0.06**	0.05**	0.04**	0.10***
Subjective knowledge	−0.00	0.00	0.01	−0.00
**Demographics**
Gender	−0.02	−0.02	0.02	−0.01
Age	−0.00	−0.00	0.01	0.00
Education	−0.08***	−0.06***	−0.07***	−0.15***
Household income	−0.01	−0.02	−0.04***	−0.05*
**Cultures**
Individualism	−0.05***	−0.05***	−0.07***	−0.13***
Egalitarianism	−0.00	−0.01	−0.01	−0.02
Fatalism	−0.02***	−0.01*	−0.02***	−0.04***
Hierarchy	0.05***	0.05***	0.06***	0.12***
COVID-19 provincial experience	−0.50***	−0.79***	−0.02	−0.97***
Constant	3.90***	3.91***	4.03***	1.75***
F significance	41.70***	70.63***	37.60***	62.38***
R^2^	0.21	0.31	0.19	28
R^2^ Adjusted	0.20	0.30	0.19	28

* *p < 0.05*;

** *p < 0.01*;

*** *p <0.001*.

Attention to news is associated with risk judgment index, negative affect, perceived risk susceptibility of the area where respondents live in, perceived risk susceptibility to self, subjective knowledge level, and individuals who are older, more educated, and have more household income (see Table 6).

**Table 6:** Linear regressions of attention to news and survey of public reactions to COVID-19, China, 2020.

	**Attentions to news**
Risk judgement index	0.06***
**Affective risk perception**
Dread of risk	−0.02
Positive affect	−0.14***
**Cognitive risk perception**
Risk susceptibility-my area	0.03*
Risk susceptibility-other area	−0.01
Personal risk susceptibility	0.05***
Likelihood of a future outbreak	−0.02
Subjective knowledge	0.17***
**Demographics**
Gender	−0.02
Age	0.08***
Education	0.08***
Household income	0.04**
**Cultures**
Individualism	−0.01
Egalitarianism	0.01
Fatalism	−0.01
Hierarchy	−0.01
COVID-19 provincial experience	0.06
Constant	4.25***
F significance	18.52***
R^2^	0.10
R^2^ Adjusted	0.10

* *p < 0.05*;

** *p < 0.01*;

*** *p < 0.001*.

## Discussions

### Main Conclusions

Potential explanatory factors, collectively, have much greater effect sizes for risk susceptibility-my area (R^2^ adjusted = 32%), trust in provincial government (30%), risk to self (28%) than risk to global (14%), likelihood of a future outbreak (12%), risk susceptibility-other area (12%), and attention to news (10%). The adjusted effect sizes for the other variables range from 15 to 25%: dread of risk (25%), personal risk susceptibility (24%), trust in local government (20%), trust in central government (19%), risk to China (17%), and positive affect (16%). The low explanation for attention to news may reflect that the common predictors used here did not take account of other possible associations (i.e., information insufficiency, information processing).

This research is not a unique systematic analysis on public reactions during a public health crisis (12), but it is still rare during the COVID-19 pandemic. As for potential explanations of different reactions to COVID-19, risk judgement index is the most frequent predictor for the four sets of dependent variables: affective risk perception, cognitive risk perception, trust in government, and attention to news. Among the risk perception variables, dread of risk, risk perception-my area, and personal risk susceptibility are also frequently predicting specific reactions to COVID-19. Consistent with prior research (11, 12, 52), the higher the cognitive and affective risk perceptions, the higher the risk judgments. By further distinguishing different dimensions of risk susceptibility, this research found that perceived risk susceptibility in other area is negatively associated with judged risk to self but positively associated with judged risk to China and global. Moreover, risk judgement, negative affect, perceived risk, and susceptibility to self and to the area where the respondents live in are positively correlated with public attentions to news. Given the cross-sectional nature of the survey data, this research cannot speak of a causal link between risk perceptions and attention to news. However, it confirmed a potential association between risk perception and information seeking in the RISP model.

Panic, which often refers to affective risk perception, is the core of concerns in risk analyses. Risk perception literature has suggested exposure to news coverage can increase risk perception, women often rate risks higher than men, and egalitarians rate risks higher than other cultures. This research also found that females and elders are more likely to dread COVID-19, and that attention to news and egalitarianism have negative effects on negative affect. However, since prior research has implied that different types of social media networks (government-controlled or official social media vs. semi-official sources vs. unofficial sources) have different effects on risk perceptions (5, 57, 58), a joint analysis of causal links among media exposure, media content, informational source, and citizen's different kinds of reactions would be required. Moreover, subjective knowledge is positively associated with positive affect and attention to news. Therefore, risk managers who want to educate the public to keep calm may interpret it as the need to better explain scientific knowledge of COVID-19. This research also found differences of risk perception among diverse demographic and cultural groups. For example, elders and males saw high personal risk but youngers saw high risk to China, and females perceive high-risk susceptibility in other area. Fatalists saw high personal risk susceptibility and egalitarians saw high-risk susceptibility in the area they live. Moreover, although living in a risky area (e.g., Hubei province) increased cognitive risk perception and risk judgments, people living in the risky area do not panic much compared to people living in other areas. Therefore, it is both theoretically and practically reasonable to distinguish among the three measures of risk perceptions.

Another intriguing finding is that trust in government did not influence affective risk perceptions. The assumption that trust in government decreases fear or risk judgments (24, 25) has been rejected at least once in existing research (10). Again, caution is warranted when using cross-sectional data. However, the findings here suggest a complex effect in which trust has positive effects (e.g., risk judgment to China and global), negative effects (e.g., cognitive risk perceptions), and no effect (e.g., two affective risk perceptions) on different measures of risk perceptions.

Turning to the potential predictors of trust in government. In general, this research saw a relatively high level of trust in government, especially high trust in the central government. This finding may reflect the “rally round the flag effect (or syndrome)” that the government experienced increased public support during the early stages of the outbreak (59) and is consistent with previous evidence that public trust increases during the early stage of the COVID-19 pandemic (44, 45). In addition to the finding that trust in government is not always negatively associated risk perceptions, this research indicated that people who are better educated and have a higher level of income are more skeptical about the government. Unlike western countries, attention to news increased trust in the three levels of government, probably because social media in China could be viewed as one of the channels of political mobilization (38). Here, the results suggest that modernization processes and political mobilization are working in opposite directions in China in influencing the public's political trust. Furthermore, except that egalitarianism is indifferent of trust in government, individualism and fatalism have a negative effect, and hierarchy has a positive effect on three levels of trust in government. Finally, living in Hubei is negatively associated trust in local/provincial government but not associated with trust in central government. These results implied that COVID-19 pandemic in Hubei has seriously harmed political trust at local level. In summary, this research suggests that to rebuild trust, political mobilization, taking care of different cultures in risk management, and governmental performance in responding to public health emergencies are all important.

### Limitations and Final Remarks

This research has three limitations: first, this web-based survey used a non-representative sample with over-representation of younger, better educated, and higher-income individuals, and future studies would benefit from confirmation with representative samples. Moreover, the cross-sectional analyses here are not able to untangle causal relationships. Longitudinal data should be used in future research. Field survey can be helpful to recruit participants who are older, less educated, and lower-income individuals. Second, measures of risk perceptions and trust can be improved. For example, risk vulnerability and severity of a negative outcome are two other common measures of risk perception. Other research distinguished trust in government and confidence in government [e.g., the trust, confidence, and cooperation model (20)], which should also be added in future research. Third, this study is confined to China, and a cross-country comparative study is needed to expand the applicability of the current findings. For example, previous comparative research has found cross-national disparity in public perceptions during the COVID-19 pandemic (10). Moreover, the previous comparative research has found that while countries with less democratic systems, such as China, imposed more stringent restrictions to control the first wave of COVID-19 and experience less mortality rate, countries that are more democratic have exceeded those with less democratic regimes regarding their testing efforts. Therefore, future research should consider the tradeoff between imposing astringent policy and sacrificing democracy in different countries.

Despite the limitations mentioned above, this study made a systematic attempt to explain public reactions when COVID-19 appeared in China. First, although risk judgments, cognitive risk perception, and affective risk perceptions are closely associated with each other, both risk managers and researchers should recognize the differences of their conceptions and potential predictors. For example, the subgroups who felt themselves or the area they live are at risk are not the same groups of people who perceive high risk of COVID-19 to the China/global/other areas. Second, trust in government may decrease risk judgment and cognitive risk perception, but trust alone has a limited role in keeping the public from experiencing negative emotions. Panic can be derived from other factors, such as risk judgment and cognitive risk perceptions, attention to news, and being female and older. However, the associations among these variables implied more complex situations that risk managers may need to deal with. For example, attention to news may increase dread of risk but yield higher level of trust in government, as social media networks are a potential way of increasing trust and mobilizing and educating the public in China. Finally, different demographic and cultural subgroups have displayed different patterns of both risk perceptions and trust in government. Risk managers and communicators should consider the varieties of reactions from these subgroups, especially those who are more likely to fear COVID-19 (e.g., elders, females, or egalitarians). As China has been reportedly getting the outbreak under control and COVID-19 has created “new normal” worldwide, some findings in this research may be transient (e.g., negative effect of COVID-19 provincial experience on trust in local/provincial government), other associations may persist (e.g., associations between culture and trust, associations among attention to news, risk perceptions, and trust).

## Data Availability Statement

The data analyzed in this study is subject to the following licenses/restrictions: The dataset is available on request. Requests to access these datasets should be directed to xgbb0203@gmail.com.

## Author Contributions

The sole author is responsible for writing—reviewing and editing, validation, conceptualization, methodology, software, writing—original draft preparation, formal analysis, and visualization.

## Funding

This work was supported by National Natural Science Foundation of China (Grant No. 72104039), Humanity and Social Science Youth foundation of Ministry of Education of China (Grant No. 21XJC810001), Chongqing University Fundamental Research Funds for the Central Universities (Grant No. 2021CD8KXYGG006), and the Chinese Postdoctoral Science Foundation (Grant No. 2021M700615).

## Conflict of Interest

The author declares that the research was conducted in the absence of any commercial or financial relationships that could be construed as a potential conflict of interest.

## Publisher's Note

All claims expressed in this article are solely those of the authors and do not necessarily represent those of their affiliated organizations, or those of the publisher, the editors and the reviewers. Any product that may be evaluated in this article, or claim that may be made by its manufacturer, is not guaranteed or endorsed by the publisher.

## References

[1] World Health Organizations (WHO) Coronavirus (COVID-19) Dashboard. Available online at: https://covid19.who.int/ (accessed March 25, 2021).

[2] Yang Z Xin Z. Heterogeneous risk perception amid the outbreak of COVID-19 in China: implications for economic confidence. Appl Psychol Health Well Being. (2020) 12:1000–18. 10.1111/aphw.12222 32875730

[3] Liu M Zhang H Huang H. Media exposure to COVID-19 information, risk perception, social and geographical proximity, and self-rated anxiety in China. BMC Public Health. (2020) 20:1649. 10.1186/s12889-020-09761-8 33148201 PMC7609828

[4] Ding Y Xu J Huang S Li P Lu C Xie S. Risk perception and repression in public health viruses: evidence from the COVID-19 crisis in China. Int J Environ Res Public Health. (2020) 17:5728. 10.3390/ijerph17165728 32784792 PMC7460398

[5] Zhong Y Liu W Lee T-Y Zhao H Ji J. Risk perception knowledge, information sources and emotional states among COVID-19 patients in Wuhan, China. Nurs Outlook. (2021) 69:13–21. 10.1016/j.outlook.2020.08.005 32980153 PMC7442898

[6] Ye M Lyu Z. Trust, risk perception, and COVID-19 infections: evidence from multilevel analyses of combined original dataset in China. Soc Sci Med. (2020) 265:113517. 10.1016/j.socscimed.2020.113517 33218890 PMC7654228

[7] Pakpour AH Liu C-h Hou W-L Chen P Li Y-P Kuo Y-J. Comparing fear of COVID-19 and preventive COVID-19 infection behaviors between Iranian and Taiwanese older people: early reaction may be a key. Front Public Health. (2021) 9:740333. 10.3389/fpubh.2021.740333 34631652 PMC8495067

[8] Sun Y Chen X Cao M Xiang T Zhang J Wang P. Will healthcare workers accept a COVID-19 vaccine when it becomes available? A cross-sectional study in China. Front Public Health. (2021) 9:664905. 10.3389/fpubh.2021.664905 34095068 PMC8172770

[9] Kahan DM Jamieson KH Landrum A Winneg K. Culturally antagonistic memes and the Zika virus: an experimental test. J Risk Res. (2017) 20:1–40. 10.1080/13669877.2016.1260631

[10] Dryhurst S Schneider CR Kerr J Freeman ALJ Recchia G van der Bles AM. Risk perceptions of COVID-19 around the world. J Risk Res. (2020) 23:994–1006. 10.1080/13669877.2020.1758193

[11] Wilson RS Zwickle A Walpole H. Developing a broadly applicable measure of risk perception. Risk Anal. (2019) 39:777–91. 10.1111/risa.13207 30278115

[12] Johnson BB. Residential location and psychological distance in Americans' risk views and behavioral intentions regarding Zika virus. Risk Anal. (2018) 38:2561–79. 10.1111/risa.13184 30176187

[13] Lu H Schuldt JP. Communicating Zika risk: using metaphor to increase perceived risk susceptibility. Risk Anal. (2018) 38:2525–34. 10.1111/risa.12982 29486059

[14] Slovic P. Perception of risk. Science. (1987) 236:280–5. 10.1126/science.3563507 3563507

[15] Mayorga MW Johnson BB. A longitudinal study of concern and judged risk: the case of Ebola in the United States, 2014–2015. J Risk Res. (2019) 22:1280–93. 10.1080/13669877.2018.1466827

[16] O'Connor RE Bard RJ Fisher A. Risk perceptions general environmental beliefs, and willingness to address climate change. Risk Anal. (1999) 19:461–71. 10.1111/j.1539-6924.1999.tb00421.x

[17] Martin IM Bender H Raish C. What motivates individuals to protect themselves from risks: the case of wildland fires. Risk Anal. (2007) 27:887–900. 10.1111/j.1539-6924.2007.00930.x 17958499

[18] Kramer AD Guillory JE Hancoc JT. Emotional contagion through social networks. Proc Natl Acad Sci USA. (2014) 111:8788–90. 10.1073/pnas.1320040111 24889601 PMC4066473

[19] Walpole HD Wilson RS. Extending a broadly applicable measure of risk perception: the case for susceptibility. J Risk Res. (2020) 24:135–47. 10.1080/13669877.2020.1749874

[20] Slovic P Peters E. Risk perception and affect. Curr Direct Psychol Sci. (2006) 15:322–5. 10.1111/j.1467-8721.2006.00461.x

[21] Slovic P. Perceived risk trust, and democracy. Risk Anal. (1993) 13:675–82. 10.1111/j.1539-6924.1993.tb01329.x

[22] Earle TC Siegrist M Gutscher H. Trust, risk perception and the TCC model of cooperation. In: Earle TC Siegrist M Gutscher H editors. Trust in Cooperative Risk Management: Uncertainty and Scepticism in the Public Mind. Verlag: Taylor & Francis Ltd. (2012). p. 1–49.

[23] Flynn J Slovic P Mertz Gender CK. race, and perception of environmental health risks. Risk Anal. (1994) 14:1101–8. 10.1111/j.1539-6924.1994.tb00082.x 7846319

[24] Johnson BB Slovic P. Fearing or fearsome Ebola communication? Keeping the public in the dark about possible post-21-day symptoms and infectiousness could backfire. Health Risk Soc. (2015) 17:458–71. 10.1080/13698575.2015.1113237

[25] Wong CML Jensen O. The paradox of trust: perceived risk and public compliance during the COVID-19 pandemic in Singapore. J Risk Res. (2020) 23:1021–30. 10.1080/13669877.2020.1756386

[26] van der Linden S Leiserowitz A Maibach E. The gateway belief model: a large-scale replication. J Environ Psychol. (2015) 62:49–58. 10.1016/j.jenvp.2019.01.009

[27] Johnson BB. Explaining Americans' responses to dread epidemics: an illustration with Ebola in late 2014. J Risk Res. (2017) 20:1338–57. 10.1080/13669877.2016.1153507

[28] Pidgeon N Kasperson RE Slovic P. The Social Amplification of Risk. Cambridge: Cambridge University Press (2003).

[29] Young ME Norman GR Humphreys KR. Medicine in the popular press: the influence of media on perceptions of disease. PLoS ONE. (2008) 3:e3552. 10.1371/journal.pone.0003552 18958167 PMC2569209

[30] Chen R-X Ge Z-M Hu S-L Tang W-Z. Supportive or confining? The impact of war metaphors from the COVID-19 pandemic on persons with disabilities in Mainland China. Front Public Health. (2021) 9:720512. 10.3389/fpubh.2021.720512 34616706 PMC8488130

[31] Douglas M Wildavky A. Risk Culture: An Essay on the Selection of Technological and Environmental Dangers. Berkeley, CA: University of California Press (1983).

[32] Johnson BB Swedlow B. Cultural Theory's contributions to risk analysis: a thematic review with directions and resources for further research. Risk Anal. (2021) 41:429–55. 10.1111/risa.13299 30970166

[33] Jones MD. Cultural characters and climate change: how heroes shape our perception of climate science. Soc Sci Quart. (2014) 95:1–39. 10.1111/ssqu.12043

[34] Xue W Marks ADG Hine DW Phillips WJ Zhao S. The new ecological paradigm and responses to climate change in China. J Risk Res. (2018) 21:323–39. 10.1080/13669877.2016.1200655

[35] Song G. Understanding public perceptions of benefits and risks of childhood vaccinations in the United States. Risk Anal. (2014) 34:541–55. 10.1111/risa.12114 24033739

[36] Johnson BB Mayorga M. Temporal shifts in Americans' risk perceptions of the Zika outbreak. Human Ecol Risk Assess. (2020) 27:1242–57. 10.1080/10807039.2020.1820852

[37] Maaravi Y Levy A Gur T Confino D Segal S. The tragedy of the commons”: how individualism and collectivism affected the spread of the COVID-19 pandemic. Front Public Health. (2021) 9:627559. 10.3389/fpubh.2021.627559 33643992 PMC7905028

[38] Eiser JR Miles S Frewer L Trust J. Perceived risk and attitudes towards food technologies. J Appl Soc Psychol. (2002) 32:2423–33. 10.1111/j.1559-1816.2002.tb01871.x

[39] Poortinga W Pidgeon F. Exploring the dimensionality of trust in risk regulation. Risk Anal. (2005) 23:199–209. 10.1111/j.0272-4332.2005.00579.x 12969411

[40] Li L. Distrust in government and preference for regime change in China. Polit Stud. (2020) 69:1–18. 10.1177/0032321719892166

[41] Kampen JK De Walle SV Bouckaert G. Assessing the relation between satisfaction with public service delivery and trust in government: the impact of the predisposition of citizens toward government on evaluations of its performance. Public Perform Manag Rev. (2006) 29:387–404. 10.1080/15309576.2006.11051881

[42] Yang Q Tang W. Exploring the sources of institutional trust in China: Culture, mobilization, or performance? Asian Polit Policy. (2010) 2:415–36. 10.1111/j.1943-0787.2010.01201.x

[43] Geddes B Zaller J. Sources of popular support for authoritarian regimes. Am J Polit Sci. (1989) 33:319–47. 10.2307/2111150

[44] Gozgor G. Global evidence on the determinants of public trust in governments during the COVID-19. Appl Res Quality Life. (2020) 1–20. 10.2139/ssrn.3618837 33564341 PMC7862976

[45] Vu VT. Public trust in government and compliance with policy during COVID-19 pandemic: empirical evidence from Vietnam. Public Organiz Rev. (2021). 10.1007/s11115-021-00566-w

[46] Tumlison C Moyer R Song G. The origin and role of trust in local policy elites' perceptions of high-voltage powerline installations in the state of Arkansas. Risk Anal. (2017) 37:1018–36. 10.1111/risa.12662 27996155

[47] Griffin RJ Dunwoody S Neuwirth K. Proposed model of the relationship of risk information seeking and processing to the development of preventive behaviors. Environ Res. (1999) 80:S230–45. 10.1006/enrs.1998.3940 10092438

[48] Lindell MK Perry RW. The protective action decision model: theoretical modifications and additional evidence. Risk Anal. (2012) 32:616–32. 10.1111/j.1539-6924.2011.01647.x 21689129

[49] Yang ZJ Kahlor L. What, we worry? The role of affect in information seeking and avoidance. Sci Commun. (2013) 35:189–212. 10.1177/107554701244187325482843

[50] Wei J Zhao M Wang F Cheng P Zhao D. An empirical study of the Volkswagen crisis in China: Customers' information processing and behavioral intentions. Risk Anal. (2016) 36:114–29. 10.1111/risa.12446 26178313

[51] Yan J Wei J Zhe O Vinnikova A Zhao D Zhang H. The influence of parents' information processing on childhood vaccine acceptance after a vaccine crisis in China. Health Risk Soc. (2019) 21:284–303. 10.1080/13698575.2019.1619672

[52] Safi AS Smith WJ Liu Z. Rural nevada and climate change: vulnerability, beliefs, risk perception. Risk Anal. (2012) 32:1041–59. 10.1111/j.1539-6924.2012.01836.x 22583075

[53] Heath A Martin J. Why are there so few formal measuring instruments in social and political research? In: Lyberg L Biemer P Collins M Leeuw ED Dippo C Schwarz N., editors. Survey Measurement and Process Quality. New York, NY: Wiley. (1997) p. 271–86.

[54] Li L. Political trust in rural China. Modern China. (2004) 2:228–58. 10.1177/0097700403261824

[55] Tang W. Populist authoritarianism: Chinese political culture and regime sustainability. J Chin Polit Sci. (2017) 22:489–90. 10.1007/s11366-017-9502-y

[56] Blair RA Morse BS Tsai LL. Public health and public trust: survey evidence from the Ebola virus disease epidemic in liberia. Soc Sci Med. (2017) 172:89–97. 10.1016/j.socscimed.2016.11.016 27914936

[57] Stockmann D. Race to the bottom: media marketization and increasing negativity toward the United States in China. Polit Commun. (2011) 28:268–90. 10.1080/10584609.2011.572447

[58] Vyncke B Perko T Van Gorp B. Information sources as explanatory variables for the Belgian health-related risk perception of the Fukushima nuclear accident. Risk Anal. (2017) 37:570–82. 10.1111/risa.12618 27322693

[59] Dempere J. A recipe to control the first wave of COVID-19: more or less democracy? Transform Governm People Proc Policy. (2021). 10.1108/TG-08-2020-0206

