# Supplementary material for: Explaining Chinese Reactions to COVID-19 During the Outbreak: A Systematic Illustration
Source: Front Public Health. 2021 Dec 8;9:727369. doi: 10.3389/fpubh.2021.727369 (PMC8692271; doi:10.3389/fpubh.2021.727369)
Supplement: Supplementary file 1 [file Table_1.DOCX]

Appendices

**Appendix A Selected Survey Instruments (Translated from Chinese)**

Start of Block: Knowledge & follow news

How much do you know about the coronavirus?

- Never heard of it
- Heard of it but don't know any more
- Know about it in general but not details
- Know some details about it
- Know a lot of details about it
- I am an expert on the coronavirus

How closely are you following news about the coronavirus infections in China?

- Not at all
- Once a month
- Once a week
- Many times a week
- Many times a day

End of Block: Knowledge & follow news

Start of Block: Risk

How much risk does the coronavirus pose to you or your family?

- No risk
- Little risk
- Slight risk
- Moderate risk
- High risk
- Very high risk

How much risk does the coronavirus pose to China?

- No risk
- Little risk
- Slight risk
- Moderate risk
- High risk
- Very high risk

How much risk does the coronavirus pose to the world?

- No risk
- Little risk
- Slight risk
- Moderate risk
- High risk
- Very high risk

How does considering the coronavirus make you feel, from very bad to very good?

- Very bad
- Somewhat bad
- Slightly bad
- Neither good nor bad
- Slightly good
- Somewhat good
- Very good

Where “dread” means to be in terror of, or fear intensely, how much do you dread the coronavirus?

- No dread
- Slight dread
- Some dread
- Moderate dread
- High dread
- Very high dread

Please answer whether you agree or disagree with the statements (1=Strongly disagree, 2=Disagree, 3=Slight disagree, 4=Slight agree, 5= Agree, 6=Strongly agree)?

- My area is one of the places that is very likely to be affected by the coronavirus.
- The coronavirus mainly affects areas far from where I live in.
- The coronavirus is most likely to have a big impact on people like me.

How likely they think there will be another large outbreak of the coronavirus in China in the next five years?

- Not at all likely
- Somewhat unlikely
- Somewhat likely
- Very likely.

End of Block: Risk

Start of Block: Political trust

Please rate how much you trust your local government (municipal or lower level government) to protect people from the coronavirus.

- No trust at all
- Slight trust
- Moderate trust
- High trust

Please rate how much you trust your provincial government to protect people from the coronavirus.

- No trust at all
- Slight trust
- Moderate trust
- High trust

Please rate how much you trust your central government to protect people from the coronavirus.

- No trust at all
- Slight trust
- Moderate trust
- High trust

End of Block: Political trust

Start of Block: Cultures

Please answer whether you agree or disagree with the statements (1=Strongly disagree, 2=Disagree, 3=Slight disagree, 4=Slight agree, 5= Agree, 6=Strongly agree)?

Egalitarianism:

- Society works best if power is shared equally.
- What society needs is a fairness revolution to make the distribution of goods more equal.
- It is our responsibility to reduce differences in income between the rich and the poor.

Hierarchy:

- Society would be much better off if the people in charge imposed strict and swift punishment on those who break the rules.
- Society is in trouble because people do not obey those in authority.
- The best way to get ahead in life is to work hard to do what you are told to do.

Individualism:

- We are all better off when we compete as individuals.
- Even the disadvantaged should have to make their own way in the world.
- Even if some people are at a disadvantage, it is best for society to let people succeed or fail on their own.

Fatalism:

- No matter how hard we try, the course of our lives is largely determined by forces beyond our control.
- The most important things that take place in life happen by chance.
- It would be pointless to make serious plans in such an uncertain world.

Note: The order of cultural statements was randomized in this section.

End of Block: Cultures

Start of Block: Demographics

What is your age?

Which option best describes your gender?

- Male
- Female
- Choose not to respond

Please indicate your highest level of education:

Junior high school and below

- High school
- Three years college
- Bachelor’s degree
- Master’s degree
- Ph.D. degree

What is your annual household income, including salaries, wages, Social Security, pensions, and all other income to members of your household?

- Below ¥50,000
- ¥50,000-¥100,000
- ¥100,000-¥200,000
- ¥200,000-¥500,000
- ¥500,000-¥1,000,000
- More than ¥1,000,000

What is the six-digit zip code for your residence?

End of Block: Demographics

**Appendix B Pairwise Correlations**

|  | (1) | (2) | (3) | (4) | (5) | (6) | (7) | (8) | (9) | (10) | (11) | (12) |
| --- | --- | --- | --- | --- | --- | --- | --- | --- | --- | --- | --- | --- |
| Risk to self(1) | 1.00 |  |  |  |  |  |  |  |  |  |  |  |
| Risk to China(2) | 0.34 | 1.00 |  |  |  |  |  |  |  |  |  |  |
| Risk to global(3) | 0.21 | 0.60 | 1.00 |  |  |  |  |  |  |  |  |  |
| Dread of risk(4) | 0.37 | 0.30 | 0.22 | 1.00 |  |  |  |  |  |  |  |  |
| Positive affect(5) | -0.17 | -0.26 | -0.19 | -0.35 | 1.00 |  |  |  |  |  |  |  |
| Risk susceptibility-my area(6) | 0.41 | 0.24 | 0.08 | 0.24 | -0.17 | 1.00 |  |  |  |  |  |  |
| Risk susceptibility-other area(7) | 0.02 | 0.14 | 0.13 | 0.09 | -0.04 | 0.11 | 1.00 |  |  |  |  |  |
| Personal risk susceptibility(8) | 0.25 | 0.17 | 0.14 | 0.25 | -0.13 | 0.35 | 0.23 | 1.00 |  |  |  |  |
| Likelihood of a future outbreak(9) | 0.16 | 0.13 | 0.14 | 0.20 | -0.09 | 0.19 | 0.15 | 0.23 | 1.00 |  |  |  |
| Trust in local government(10) | -0.12 | 0.04 | 0.15 | -0.04 | -0.02 | -0.20 | 0.06 | -0.10 | -0.09 | 1.00 |  |  |
| Trust in provincial government(11) | -0.13 | 0.04 | 0.16 | -0.06 | 0.01 | -0.25 | 0.11 | -0.10 | -0.11 | 0.81 | 1.00 |  |
| Trust in central government(12) | -0.03 | 0.04 | 0.11 | -0.01 | 0.00 | -0.06 | 0.01 | -0.06 | -0.13 | 0.44 | 0.46 | 1.00 |
| Attentions to news(13) | 0.12 | 0.14 | 0.09 | 0.08 | -0.15 | 0.14 | 0.02 | 0.12 | 0.03 | 0.03 | 0.01 | 0.03 |
| Subjective knowledge(14) | 0.03 | 0.02 | 0.03 | -0.01 | 0.03 | 0.03 | 0.01 | -0.01 | -0.04 | -0.01 | 0.00 | 0.00 |
| Gender(15) | 0.03 | -0.02 | 0.02 | -0.08 | 0.03 | 0.00 | -0.01 | 0.09 | 0.06 | -0.03 | -0.03 | -0.02 |
| Age(16) | 0.15 | 0.00 | 0.10 | 0.09 | -0.07 | 0.00 | 0.05 | 0.12 | 0.13 | 0.04 | 0.04 | 0.06 |
| Education(17) | -0.03 | -0.04 | -0.17 | -0.03 | 0.00 | 0.15 | -0.04 | -0.07 | 0.00 | -0.18 | -0.18 | -0.20 |
| Household income(18) | 0.05 | -0.03 | -0.08 | 0.00 | 0.02 | 0.13 | 0.03 | 0.01 | 0.05 | -0.11 | -0.12 | -0.16 |
| Individualism(19) | -0.01 | -0.02 | -0.04 | 0.00 | 0.03 | 0.04 | 0.01 | 0.06 | 0.08 | -0.21 | -0.19 | -0.30 |
| Egalitarianism(20) | 0.04 | 0.09 | 0.10 | 0.07 | -0.09 | 0.07 | 0.02 | 0.09 | 0.07 | 0.00 | -0.01 | 0.00 |
| Fatalism(21) | 0.00 | 0.02 | 0.05 | 0.06 | -0.01 | 0.01 | 0.08 | 0.17 | 0.14 | -0.10 | -0.07 | -0.13 |
| Hierarchy(22) | 0.02 | 0.11 | 0.14 | 0.07 | -0.04 | -0.01 | 0.04 | 0.03 | -0.03 | 0.18 | 0.18 | 0.25 |
| COVID-19 provincial experience(23) | 0.26 | 0.00 | -0.09 | 0.06 | -0.04 | 0.36 | -0.20 | 0.09 | 0.02 | -0.32 | -0.47 | -0.05 |

(Continued on following page)

**Appendix B Pairwise Correlations (Continued)**

|  | (13) | (14) | (15) | (16) | (17) | (18) | (19) | (20) | (21) | (22) | (23) |
| --- | --- | --- | --- | --- | --- | --- | --- | --- | --- | --- | --- |
| Attentions to news(13) | 1.00 |  |  |  |  |  |  |  |  |  |  |
| Subjective knowledge(14) | 0.18 | 1.00 |  |  |  |  |  |  |  |  |  |
| Gender(15) | 0.02 | 0.04 | 1.00 |  |  |  |  |  |  |  |  |
| Age(16) | 0.09 | -0.08 | 0.23 | 1.00 |  |  |  |  |  |  |  |
| Education(17) | 0.07 | 0.06 | -0.07 | -0.38 | 1.00 |  |  |  |  |  |  |
| Household income(18) | 0.10 | 0.11 | 0.06 | 0.00 | 0.35 | 1.00 |  |  |  |  |  |
| Individualism(19) | -0.02 | 0.02 | 0.11 | 0.01 | 0.09 | 0.09 | 1.00 |  |  |  |  |
| Egalitarianism(20) | 0.05 | 0.00 | 0.12 | 0.21 | -0.09 | -0.07 | 0.13 | 1.00 |  |  |  |
| Fatalism(21) | -0.03 | -0.05 | 0.03 | 0.09 | -0.12 | -0.05 | 0.33 | 0.16 | 1.00 |  |  |
| Hierarchy(22) | 0.00 | -0.01 | -0.01 | 0.08 | -0.18 | -0.11 | -0.01 | 0.29 | 0.11 | 1.00 |  |
| COVID-19 provincial experience(23) | 0.06 | 0.01 | 0.00 | -0.03 | 0.11 | 0.07 | -0.01 | 0.01 | -0.04 | -0.04 | 1.00 |

**Appendix C Descriptive Statistics**

| Variable | N | Mean | Std. Dev. | Min | Max |
| --- | --- | --- | --- | --- | --- |
| *Risk judgment* | | | | | |
| Risk to self | 2863 | 3.65 | 1.31 | 1 | 6 |
| Risk to China | 2863 | 4.92 | 0.83 | 1 | 6 |
| Risk to global | 2863 | 4.65 | 0.99 | 1 | 6 |
| *Affective risk perception* | | | | | |
| Dread of risk | 2863 | 3.42 | 1.26 | 1 | 6 |
| Positive affect | 2863 | 1.59 | 0.73 | 1 | 5 |
| *Cognitive risk perception* | | | | | |
| Risk susceptibility-my area | 2863 | 4.43 | 1.31 | 1 | 6 |
| Risk susceptibility-other area | 2863 | 3.72 | 1.27 | 1 | 6 |
| Personal risk susceptibility | 2863 | 3.85 | 1.32 | 1 | 6 |
| Likelihood of a future outbreak | 2863 | 2.66 | 0.73 | 1 | 4 |
| *Trust in government* | | | | | |
| Trust in local government | 2863 | 2.99 | 0.82 | 1 | 4 |
| Trust in provincial government | 2863 | 2.3 | 0.86 | 1 | 4 |
| Trust in central government | 2863 | 3.47 | 0.68 | 1 | 4 |
| *Attention to news* | 2863 | 5.42 | 0.83 | 1 | 6 |
| *Subjective Knowledge* | 2863 | 3.88 | 0.82 | 1 | 6 |
| *Demographics* | | | | | |
| Age | 2863 | 2.69 | 1.23 | 1 | 6 |
| Education | 2863 | 3.95 | 1.14 | 1 | 6 |
| Household income | 2863 | 2.64 | 1.13 | 1 | 6 |
| *Cultures* | | | | | |
| Individualism | 2863 | 9.22 | 2.43 | 3 | 18 |
| Egalitarianism | 2863 | 12.76 | 2.6 | 3 | 18 |
| Fatalism | 2863 | 9.86 | 2.73 | 3 | 19 |
| Hierarchy | 2863 | 11.88 | 2.53 | 3 | 18 |
